# Evaluation of Association Between the Serum Levels of MMP-9 and MMP-9/TIMPs With Soluble Forms of Selectins and Itching Induced by Sulfur Mustard

**Published:** 2017-01-27

**Authors:** Nayere Askari, Tooba Ghazanfari, Mohammad Mehdi Naghizadeh, Athar Moin, Ali Khamesipour, Shahryar Pourfarzam, Zuhair Mohammad Hassan

**Affiliations:** 1 *Dept. of Biology, Faculty of Basic Sciences, Shahid Bahonar University of Kerman, Kerman, Iran*; 2 *Immunoregulation Research Center, Shahed University, Tehran, Iran*; 3 *Center for Research and Training in Skin Diseases and Leprosy, Tehran University of Medical Sciences, * *Tehran, Iran*; 4 *Dept. of Immunology, Tarbiat Moddares University, Tehran, Iran*

**Keywords:** Mustard Gas, Pruritus, MMP-9, MMP-9/TIMPs, Selectins

## Abstract

**Background & objective:**

Pruritus is the most frequent chronic dermal complication of sulfur mustard (SM), which negatively influences the quality of life. Exact pathophysiology of SM-induced itching is unknown. The current study aimed at evaluating the possible association between SM-induced itching and the serum levels of matrix metalloproteinase (MMP)-9 and their endogenous inhibitors, and serum levels of soluble forms of selectins (sL-, sP-, and sE-selectins) as adhesion molecules involved in the development of different inflammatory reactions.

**Methods:**

Serum levels of MMP-9, MMP-9/ tissue inhibitors of metalloproteinases (TIMPs), and selectins were measured by the enzyme-linked immunosorbent assay (ELISA), and compared between the groups (n=368) with and without itching, and matched control groups (n=126).

**Results:**

Serum levels of MMP-9 were significantly higher in the SM exposed group with itching, compared with that of the group without itching (medians: 894 and 624 pg/mL respectively; P-value =0.034). There was no relationship between the serum levels of MMP-9/TIMP-1, MMP-9/TIMP-2, MMP-9/TIMP-4, and itching in the patients exposed to SM. Median serum levels of sE- and sL-selectins in the exposed group with itching were higher than those of the exposed group without itching. These differences were statistically insigniﬁcant (P-values =0.084 and 0.095, respectively).

**Conclusion:**

According to the results of the current study, the increased serum levels of MMP-9 and selectins 20 years after exposure may play role in the pathogenesis and persistence of SM-induced itching in the exposed individuals.

## Introduction

Exposure to sulfur mustard (SM) leads to short- and long-term adverse effects in various organs including the skin. Skin is the first and most heavily damaged organ upon SM exposure. In Sardasht-Iran Cohort study (SICS), the most common skin symptom was pruritus and the difference between the exposed and control groups was statistically significant (1). The prevalence of itching in the studies by Moin et al. (1), Fekri and Janghorbani (2), Toosi et al. (3), Hefazi et al. (4), and Heidari et al., (5) was 94.7%, 54.8%, 75.1%, 65%, and 25.6%, respectively. In addition, pruritus can significantly impair patients’ quality of life (6-8). Exact pathophysiology of SM-induced itching is unknown. Drugs are used symptomatically based on individual cases (9). Chronic itch represents a burdensome clinical problem that can be resulted from various etiologies (10). The neurophysiology of pruritus is not fully understood. However, it is known that Aδ and C fibres are responsible for transmission of pruritus (11). There are numerous mediators capable of stimulating these afferent nerves resulting in itch, including biogenic amines, proteases, cytokines, and peptides (10). Inflammation is a key component in the pathogenesis of SM-induced skin complications (12). Cytokines and chemokines are inflammatory mediators and important activators of sensory nerves, thereby contributing to neurogenic inflammation, pain, and pruritus (13). Various studies revealed the role of selected matrix metalloproteinases (MMPs) in pathogenesis of several skin diseases such as dermatitis herpetiformis (14), atopic dermatitis (15), and systemic scleroderma (16). Several MMPs were previously reported to have elevated in psoriatic lesions (17, 18). MMPs hydrolyze most components of the extracellular matrix. The activities of MMPs are also controlled by natural tissue inhibitors of metalloproteinases (TIMPs). These proteinases play a central role in various biological processes, including normal tissue remodeling, wound healing, and angiogenesis (19).

In addition, adhesion molecules appear to play a role in the occurrence and development of pruritus, at least in certain dermatologic disorders. For example, selectins play a key role in the pathogenesis of common inflammatory skin disorders such as atopic dermatitis and psoriasis (20-22).

The role of MMPs and selectins in Skin SM induced disorders is poorly understood. To clarify the mechanisms and possible mediators involved in the pruritus in the patients exposed to SM, the present study aimed at assessing the serum levels of soluble forms of selectins (L, P, and E), MMP-9, and MMP-9/TIMPs complex in the SM exposed and matched control groups.

## Materials and Methods

**Study Design and Participant**

The current study was a part of SICS and the details of methodology were previously reported (23). Brieﬂy, 368 male subjects aged 20 to 60 years exposed to SM about 20 years ago, and 126 age/gender-matched unexposed volunteers were included in the cohort. SICS was initiated in 2006 and the clinical evaluation and sample collection were done in June 2007 and the experiments were completed in six months. Clinical evaluation was performed on each volunteer participant; every volunteer was interviewed and physically examined by a dermatologist. Patients exposed to SM had multiple organ complications and were categorized into subgroups to make easier understanding of any possible relationship between serum levels of MMP-9, MMP-9/TIMPs, and selectins with skin itching. In the current study, with respect to itching, the participants were divided into four groups as follows:

1. Patients exposed to SM with skin itching, 2) Patients exposed to SM without skin itching, 3) The control group with skin itching, and 4) The control group without skin itching.

**Ethical Considerations**

The study was approved by the Ethical Committee Board of Research Ethics of Janbazan Medical and Engineering Research Center (JMERC), and Board of Research, Iranian Ministry of Health and Medical Education, and Ethical committee of Shahed University. Potential volunteers were informed about the purpose and procedure of the study; the volunteers who signed an informed consent were recruited.

**Serum Collection**

Peripheral blood samples were drawn into vacutainer tubes (BD Biosciences). The sera were separated by centrifugation at 2000×g at 4°C for 20 minutes and stored at −80°C until laboratory measurements.

**Enzyme-linked Immunosorbent Assay Measurements**

Human MMP-9, MMP-9-TIMPs complex DuoSet® ELISA Development Kits (R&D Systems) were used to measure the components in the sera. Human sL-selectin/CD62L, sE-selectin/CD62E, and sP-selectin/CD62P Quantikine® ELISA kits (R&D Systems) were used to measure the selectin levels in the sera according to the manufacturer's instruction. This assay employs the quantitative sandwich enzyme immunoassay technique. The ELISA reader and washer were Stat-Fax 2100 and Stat-Fax 2600 (USA), respectively.

**Statistical Analysis**

Statistical comparison among groups was performed using Mann–Whitney test. Differences were considered statistically significant at P ≤0.05. Data were presented as mean (standard deviation) and median (first and third quartiles). Analysis of all the data was performed using SPSS software version 16.0 (Chicago, Illinois, USA).

## Results

In SICS study, 94.7% (n=338) of the exposed group and 63.3% (n=81) of unexposed control group had skin itching. There was a statistically significant difference between the exposed and matched control groups in terms of itching (P-value <0.001).

**Comparison of the serum levels of MMP-9, MMP-9/TIMP-1, MMP-9/TIMP-2 and MMP-9/TIMP-4 between the patients exposed to SM with or without itching and the matched controls**

Median serum levels of MMP-9 were 624 ng/mL in the exposed group without itching and 894 ng/mL in the exposed group with itching, 855 ng/mL in the control group with itching, and 848 ng/mL in the control group without itching. The difference between the exposed groups with and without itching was statistically significant (P-value =0.034) (Table 1).

As shown in tables 2, 3, and 4 there were no significant differences among the serum levels of MMP-9/TIMP-1, MMP-9/TIMP-2, and MMP-9/TIMP-4 between the exposed groups with without itching.

A significant increase in MMP-9/TIMP-2 complex was observed in the control group with itching compared to the control group without itching (P-value <0.008). Also, there was a significant difference between the SM-exposed group with itching and the control group (P-value=0.019) (Table 3).

**Table 1:** MMP-9 in the Control and Sulfur Mustard Exposed Groups With Itching

MMP-9 Serum (µg/mL)									
Study groups	Itching	N	Mean	SD	Median	Q_1_	Q_3_	P-value^1^	P-value^2^
Control	No	45	1.221	0.926	0.848	0.543	1.694	0.902	0.196
	Yes	78	1.025	0.599	0.855	0.630	1.179		0.315
SM-Exposed	No	20	0.889	0.642	0.624	0.465	1.231	0.034	
	Yes	320	1.772	2.357	0.894	0.646	1.718		

The serum levels of MMP-9 in volunteers with and without itching were assessed and a comparison was made between the control and exposed groups, and within each groups.

P-value1: Comparison of the serum level between participants with and without itching within each group (Mann–Whitney test).

P-value2: Comparison between the exposed groups and control groups (Mann–Whitney test). Bold data shows significant differences (P-value <0.05).

**Table 2:** MMP-9/TIMP-1 Complex in the Control and Sulfur Mustard Exposed Groups With Itching

MMP-9/TIMP-1 Serum (µg/mL)										
Study Groups	Itching	N	Mean	SD	Median	Q_1_	Q_3_	P-value^1^	P-value^2^
control	No	45	35.157	43.506	18.552	9.610	51.886	0.607	0.909
	Yes	76	27.324	28.195	18.049	7.781	32.094		0.272
Exposed	No	20	32.327	34.651	16.546	1.1017	37.834	0.850	
	Yes	317	32.263	41.168	20.646	10.032	39.898		

P-value2: Comparison between exposed subjects and corresponding controls (Mann–Whitney test). Bold data shows significant differences at P-value <0.05.

**Table 3:** MMP-9/TIMP-2 Complex in the Control and Sulfur Mustard Exposed Groups With Itching

				MMP-9/TIMP-2 Serum (µg/mL)						
Study Groups	Itching	N	Mean		SD	Median	Q_1_	Q_3_	P-value^1^	P-value^2^
Control	No	42	2.256		9.174	0.023	0.00	0.196	0.008	0.019
	Yes	78	2.935		16.726	0.246	0.041	0.844		0.859
SM-Exposed	No	20	1.343		2.919	0.439	0.00	1.194	0.272	
	Yes	316	1.681		10.520	0.271	0.00	0.682		

The serum levels of MMP-9/TIMP-2 complex in volunteers with and without itching were assessed and a comparison was made between the control and exposed groups, and within each group.

P-value1: Comparison between participants with and without itching within each group (Mann–Whitney test).

P-value2: Comparison between exposed subjects and corresponding controls (Mann–Whitney test). Bold data shows significant differences at P-value <0.05

A significant increase was observed in MMP-9/TIMP-4 complex in the exposed group without itching, compared with the matched control group (P-value <0.034) (Table 4).

**Table 4:** MMP-9/TIMP-4 complex (Serum) in control and exposed groups With itching

MMP-9/TIMP-4 Serum (ng/mL)										
Study Groups	Itching	N	Mean	SD	Median	Q_1_	Q_3_	P-value^1^		P-value^2^
Control	No	44	58.067	133.495	18.834	15.031	26.072	0.164		0.034
	Yes	76	89.335	229.037	21.042	15.667	40.491	-		0.550
Exposed	No	20	108.807	347.044	24.816	21.042	37.347	0.467		-
	Yes	315	86.626	255.396	24.502	16.302	40.79	-		-

The serum levels of MMP-9/TIMP-4 complex in volunteers with and without itching were assessed and a comparison was made between the control and exposed groups, and within each group.

P-value1: Comparison of the serum level between participants with and without itching within each group (Mann–Whitney test).

P-value2: Comparison between the patients exposed to SM and the corresponding controls (Mann–Whitney test). Bold data shows the significant differences at P-value <0.05.

**Table 5:** sE-Selectin in the Control and Sulfur Mustard Exposed Groups With Itching

Study Groups	Itching	SE-Selectin (pg/mL)						P-value^1^	P-value^2^	P-value^3^
		N	Median	Q1	Q3	Mean	SD			
Control	No	45	23.88	11.81	28.44	22	12.435	0.150	0.909	0.004
	Yes	77	24.57	17.01	32.82	26.587	14.925		0.124	
SM-Exposed	No	20	21.695	10.2	31.83	23.94	15.869	0.084	-	
	Yes	319	26.56	17.33	40.09	30.483	17.685		-	

The serum levels of sE-Selectin in volunteers with and without itching were assessed and a comparison was made between the control and SM-exposed groups, and within each group.

P-value1: Comparison between with and without itching within each group (Mann–Whitney test).

P-value2: Comparison of the SM-exposed groups with the corresponding controls (Mann–Whitney test).

P-value3: Comparison between the SM-exposed group with itching and the control group without itching (Mann–Whitney test). Bold data shows significant differences at P-value <0.05.

**Table 6:** sL-Selectin in Control and Sulfur Mustard Exposed Groups With Itching

Study Groups	Itching	SL-Selectin(pg/mL)						P-value^1^	P-value^2^
		N	Median	Q1	Q3	Mean	SD		
Control	No	45	12.360	9.547	13.850	11.919	3.134	0.791	0.082 0.062
	Yes	77	11.820	10.140	13.750	12.203	2.98		
Exposed	No	20	9.561	8.314	11.815	10.496	2.837	0.095	
	Yes	318	11.330	9.180	13.410	11.508	3.095		

The serum levels of sL-Selectin in volunteers with and without itching were assessed and a comparison was made between the control and SM-exposed groups, and within each group.

P-value1: Comparison between the participants with and without itching within each group (Mann–Whitney test).

P-value2: Comparison of the SM-exposed subjects with the corresponding controls (Mann–Whitney test). Bold data shows significant differences at P-value <0.05.

**Comparison of the Serum levels of sE-, sL-, and sP-selectins between the patients exposed to SM with or without itching and the matched controls**

Median serum levels of sE-selectin in the exposed group with itching (26.56 pg/mL) were higher than those of the SM-exposed group without itching (21.695 pg/mL). This difference was statistically insigniﬁcant (P-value=0.084). There was no significant difference between the serum levels of sE-selectin in the SM-exposed groups without itching and the matched control groups (Table 5).

 A significant increase in sE-selectin was observed between the SM-exposed group with itching and the control group without itching (P-value <0.004). As illustrated in Table 6, the serum levels of sL-selectin was lower in the SM-exposed group without itching compared with those of the SM-exposed group with itching (9.561 vs. 11.33 pg/mL, respectively). 

The difference was statistically insigniﬁcant (P-value =0.095). Also, there was a difference between the subjects exposed to SM without itching and the matched controls, but this difference was statistically insigniﬁcant (P-value =0.082). As indicated in Table 7, the serum levels of sP-selectin were significantly lower in the SM-exposed group with and without itching, compared with their matched control groups (144.22 vs. 152.32 pg/mL, P-value =0.013 for with itching and 126.67 vs. 163.3, P-value <0.031 for without itching groups). The difference between the SM-exposed groups with and without itching was statistically insignificant (Table 7).

**Table 7:** sP-Selectin in the Control and Sulfur Mustard Exposed Groups With Itching

**Study Groups**	**Itching**	**SP-Selectin (pg/mL** **)**						**P-value** ^1^	**P-value** ^2^
		N	Median	Q1	Q3	Mean	SD		
**Control**	No	45	163.3	128.9	204	167.632	55.18	0.779	0.031 0.013
	Yes	77	152.32	126.88	217.6	173.286	60.718		
**Exposed**	No	20	126.67	83.83	173.29	134.957	55.698	0.147	
	Yes	319	144.22	116.52	178.34	152.299	50.134		

The serum levels of sP-Selectin in volunteers with and without itching were assessed and a comparison was made between the control and SM-exposed groups, and within each group.

P-value1: Comparison between the participants with and without within each group (Mann–Whitney test).

P-value2: Comparison of the subjected exposed to SM with the corresponding controls (Mann–Whitney test). Bold data shows significant differences at P-value <0.05.

## Discussion

Exposure to SM leads to many delayed skin complications including itching. In SICS, the incidence of itching was reported 94.7% in the SM-exposed group, 20 years after the exposure. Exact pathophysiology of SM-induced itching is unknown. 

The current study found that even after a long post-exposure period in the patients exposed to SM, an increased serum level of MMP-9 was associated with the itching. According to the obtained results, it is suggested that the SM-exposed group without itching could have overcome the SM-induced changes that cause itching through decrease in MMP-9, and in fact this effect is compensatory, whereas, in the SM-exposed subjects with itching, MMP-9 levels were not significantly different from in the ones in the control groups.

The serum levels of MMP-9/TIMP-2, and MMP-9/TIMP-4 were different between the SM-exposed individuals and the control ones, regardless of skin itching. However, the current study results did not show signiﬁcant association between the serum levels of MMP-9/TIMP-2 and MMP-9/TIMP-4 with itching in the patients exposed to SM. In the unexposed subjects with itching, the study results did not show any signiﬁcant differences in the serum levels of MMP-9, compared with those of the healthy controls, but increase in MMP-9/TIMP-2 remained significantly in association with itching.

The data suggested that MMP-9 contributed to itching in both groups; in the SM-exposed group with different expression of *MMP-9* and in the unexposed group with an imbalance between active MMP-9 and TIMP-2. Overexpression and activation of *MMPs*, or an imbalance between active MMPs and TIMPs were suggested as key factors involved in the breakdown of extracellular matrix found in a number of the disease states (24-26). 

The mean level of MMP-9 in the serum of dry skin patients exposed to SM showed significant increase, compared with that of the normal individuals (27). Previous studies showed that the average amount of MMP-8 and -9, and TIMP-1 and -2 in the skin tissue sample of the mustard gas exposed victims was significantly higher than that of the normal individuals (28). Shakarjian et al., demonstrated the significant increase in the expression of *MMP-9* in mouse skin after SM exposure (29). *MMP-9* expression was also observed in inflammation cells and perilesional skin in bullous pemphigoid diseases, which pathologically resemble SM-induced skin lesion (30).

Epidermal hyper innervation observed in the dermatoses with intractable pruritus, such as atopic dermatitis, suggested that the hyper innervation was partially responsible for abnormal itch perception (31, 32). Neuronal MMPs also play a role in the penetration of nerve fibres into the extracellular matrix (33). The role of MMPs in SM-induced itching is poorly understood and further studies should be conducted to discover the meaning of the differential expression of *MMP-9* gene in the exposed patients with itching.

In addition, an increased serum concentration of E- and L-selectins was found in the subjects exposed to SM with itching. The difference between the patients exposed to SM with and without itching were at the borderline level of significance (P-values = 0.084 and 0.095, respectively, for E- and L-selectins). It was also shown that the level of E-selectin in the SM-exposed group with itching was significantly higher than that of the normal control group.

Unfortunately, there are currently limited data on the possible role of adhesion molecules in SM-induced skin disorders and pruritus, but various eye, skin, and lung diseases are also associated with the induction of soluble selectins (34-36). Atopic dermatitis (AD) is a chronic inflammatory skin disease characterized by an intensely pruritic skin rash (37). Gutge sell et al,. reported a statistically significant positive correlation between sE-selectin and disease activity in atopic dermatitis (21). Skin biopsies from inflamed skin of the patients with AD showed that upregulation of endothelial cell expression of *P- and E-selectin* (38), and the immunological changes in the patients with AD treated with cyclosporine included eosinophil count reduction and lower levels of E-selectin (39). Shimada et al., suggested that the elevated sL-selectin levels and abnormal *L-selectin* expression on some leukocyte subsets in patients with AD had correlations with AD-associated inflammation and that the serum level of sL-selectin was a serologic indicator of disease severity in AD (40). Lesional skin biopsies from patients with pruritus in psoriasis vulgaris showed overexpression of *E-selectin* on vascular endothelial cells. A significant correlation was observed between the severity of pruritus and the density of E-selectin immune reactive vessels (41). Due to their involvement in the pathogenesis of various local and systemic inflammatory disorders, selectins are now considered as potential diagnostic and therapeutic tools (42).

## Conclusion

The current study concluded that increased serum levels of MMP-9 and selectins 20 years after exposure to SM may have roles in pathogenesis and persistence of SM-induced itching in the exposed individuals. The mechanism of skin SM-induced itching is poorly understood. However, the similarities in altered MMP-9 and selectins levels between the patients exposed to SM with itching and other associated pathological complications could enable clinicians to understand the disease process and make better therapeutic decisions. It seems that further research is required to clarify the role of immunological parameters in the pathogenesis of SM-induced itching. However, it should be noted that exposure to SM causes alterations in the expression of various parameters of immune responses, and on the other hand, increase and decrease of SM induced immune mediators may cause disorders, whereas, some of them are considered as compensatory responses to prevent disorders. Therefore, it seems that the multifactorial mechanism of itching and achieving the desired results should be investigated further. 
